# Prognostic Importance of Lactate and Blood Gas Parameters in Predicting Mortality in Patients with Critical Malignancies

**DOI:** 10.4314/ejhs.v33i2.10

**Published:** 2023-03

**Authors:** Ahmet Erdur, Ramazan Guven, Doganay Can, Talha Tuleyb Gurkan, Ertugrul Ak, Akkan Avci

**Affiliations:** 1 Health Science University, T.R. Ministry of Health Basaksehir Cam and Sakura City Hospital, Department of Emergency Medicine, Istanbul, Turkey; 2 Health Sciences University, Kanuni Training and Research Hospital, Department of Emergency Medicine, Istanbul, Turkey; 3 Health Sciences University, Adana City Research and Training Hospital, Department of Emergency Medicine, Adana, Turkey

**Keywords:** Lactate, mortality, malignancy, emergency, blood, gases

## Abstract

**Background:**

The aim of the present study was to detect the prognostic importance of lactate and other blood gas parameters for mortality prediction in patients with critical malignancies referring to the emergency service. The general condition of patients with malignancy who have referred to the emergency department should be evaluated and it should be shown that they are not in any oncological emergency. It is a highly significant predictor of mortality after sepsis and shock in hyperlactatemia accompanying metabolic acidosis. It is significantly used for treatment monitoring.

**Methods:**

This study was planned prospective and observational study. The patients enrolled were divided into two groups including survivor and non-survivor depending on 30-day mortality. The primary outcome of the study was determined as following the mortality within 30 days.

**Results:**

The mean lactate level was 1.9 (1.4–2.5) mmol/L in the survivor group, and 2.6 (1.9–4.4) mmol/L in the non-survivor group; a significant difference was obtained between both groups (p<0.001). When the cut-off value of the lactate was determined as >2.95 mmol/L in order to differentiate the survivors from non-survivors, the sensitivity and specificity were detected as 35.0% and 86.1%, respectively. It was detected by the multivariate regression analysis that lactate predicts the 30-day mortality with a higher significance level in patients with critical malignancies.

**Conclusions:**

It was concluded that lactate is a good predictor and may be used safely in predicting 30-day mortality in patients with any critical malignancy referring to the emergency department.

## Introduction

Cancer has become the second most common cause of death for human kind in the developed world. In the past, it was considered a rare or less important problem in developing nations. Now, cancer is one of the leading causes of death in developing countries, killing more people globally each year than AIDS, tuberculosis or malaria. More than half of all new cancer cases and over 60% of the deaths occur in the poorer regions of the world (1). According to the European Cancer Information System (ECIS) European Union data of 2021, 1.2 million people in Eastern Europe, including 632,000 women and 636,000 men, were newly diagnosed with cancer in 2020 (2). Of the people who were followed-up with due to a cancer diagnosis, 691,000 died within the same year. Cancer cases increase gradually and they are refer to the hospitals' emergency services (3,4).

The general condition of patients with malignancy who have been referred to the emergency department should be evaluated to confirm they are not in an oncological emergency. Oncological emergencies are classified according to the severity of the conditions or how the tissues and organs affected (3). Oncological emergencies may include anaphylaxis, cardiac tamponade, pericardial effusion, pleural effusion, vena cava superior syndrome, tumor lysis syndrome, febrile neutropenia, malignant epidural spinal cord compression, inappropriate antidiuretic hormone syndrome, hypercalcemia, hypoglycemia, hyponatremia, lactic acidosis, hyper-viscosity syndrome and leukostasis, cytokine release syndrome, increased intracranial pressure syndrome, seizures, thromboembolism, sepsis, hemoptysis, ileus, infections, pathological fractures, chemotherapy extravasation, and engraftment syndrome (3,4).

‘Critically ill’ is a broad term used for patients who need rapid medical treatment and who have higher mortality and morbidity (3,5). These patients should be monitored carefully (4). Vital signs are usually stable. The diagnosis and treatment stages should be fast. These patients are admitted to the red triage zone of the emergency medicine clinic. Numerous classifications have been developed to identify these patients (5).

The blood gas analysis is commonly preferred in the emergency medicine clinics due to its fast and cost-effective use (6,7). Although this test's diagnostic value is lower, it helps for differential diagnosis of many diseases (5,6,8). It has an important role in evaluating critically ill patients and in showing the diseases' etiology and severity (7).

The plasma lactate level indicates tissue hypoxia (8). Many studies have reported a correlation between lactate levels and oxygen deficit in tissues (9,10). The use of lactate has come to the forefront to determine the prognosis by showing cellular hypoxia and impaired tissue circulation in certain conditions such as shock, which impairs the circulation (11–13). It is a highly significant predictor of mortality after sepsis and shock in hyperlactatemia accompanying metabolic acidosis (10,14,15). It is significantly used for treatment monitoring (16,17).

The present study aimed to detect the prognostic importance of lactate and other blood gas parameters for 30-day mortality prediction in patients with critical malignancies who were referred to emergency services.

## Methods and Materials

**Study design**: The Institutional Review Board of Kanuni Sultan Suleyman Research and Training Hospital in Istanbul, Turkey (No. 2020-KSSH-07155) approved this prospective, multi-centered, observational study. All patients provided consent forms. This prospective and observational study included adult patients with critical malignancies who have been referred to emergency departments of two training and research hospitals between July 20, 2020 and February 20, 2021. The qSOFA criteria were used to identify critically ill patients (6). The qSOFA has 3 criteria, including impaired consciousness, respiration count at or above 22, and systolic blood pressure below 100 mm-Hg. The diagnosis of critically ill patients was made with the presence of at least two of these three criteria in this study. The study's primary outcome was detected as developing mortality after a 30-day follow-up. Accordingly, the patients included in the study were divided into two groups; those who developed mortality constituted the non-survivor group, and those who did not develop mortality during the 30-day follow-up group constituted the survivor group. The patients whose malignancy diagnosis was not documented or at the stage of diagnosis, pregnant women, patients with malignancy who have been referred to the emergency department with trauma, trauma patients who have been referred to the emergency department with acute cerebrovascular accident, patients with blood gas clots, patients who could not be followed-up for 30 days, those who were not admitted to the service/intensive care unit or were admitted voluntarily, and patients who did not want to be admitted were excluded from the study. In addition, conditions due to certain drugs, chemicals, toxic components, or genetic disorders that may cause lactate accumulation were excluded from the study. Demographic data, vital signs at admission to the emergency medicine clinic, comorbidities, and venous blood gas parameters of the patients who met the study's inclusion criteria were noted in the study form. The study's blood gas samples were obtained from venous veins in the antecubital or dorsal region of the hand. These samples were analyzed by Rapidlab 1200 series or Radiometer ABL90 Flex devices.

**Statistical analysis**: The data collected from patients were evaluated with SPSS software (v24.0; IBM, Armonk, New York) (Statistical Package for the Social Sciences) and MedCalc Statistical Software (v15.2.2; MedCalc Software bvba, Ostend, Belgium). Normally distributed values were calculated as parametric tests and mean ± Standard Deviation (SD), whereas non-normally distributed values were calculated as median (50%; quartiles 25%; 75%). Categorical variables were analyzed through chi-square and Fisher's exact test. Conformity of continuous variables to normal distribution was examined via Kolmogorov-Smirnov. Student-t test was used for the normal distribution parameters, whereas Mann-Whitney U test was used for the non-normally distributed parameters for comparison of blood gas parameters with and without mortality. Univariate and multivariate regression analyses were used to identify predictors of parameters on mortality. The receiver operating characteristic (ROC) analysis was performed to determine the prognosis level of lactate because of the statistically significant association between lactate and mortality; and the area under the curve, appropriate sensitivity and specificity, and appropriate predictive value were determined. Any p value below 0.05 (p<0.05) was accepted as statistically significant. The MedCalc program was utilized to create the figures of the data.

**Ethical Statement:** This prospective, multi-centered, observational study was approved by the Institutional Review Board of Kanuni Sultan Suleyman Research and Training Hospital (No. 2020-KSSH-07155). Informed consent forms were obtained from all patients.

## Results

The median age of the 1,044 patients, including 615 males (58.9%) and 429 females (41.1%) whose data were evaluated was 66.0 (57.0–76.0) years. Although 39.9% (n=417) of these patients included in the study constituted the non-survivors group, 60.0% (n=627) of them were in the survivors group. The primary malignancy rates of the patients included in the study were lung cancer by 18.0%, colon cancer by 15.0%, gynecological malignancies by 8.9%, pancreatic cancer by 7.8%, prostate cancer by 7.5%, hematological malignancies by 6.9%, breast cancer by 6.4%, brain cancer by 6.4%, gastric cancer by 4.9%, and other malignancies by 18.2%. Table 1 presents the distribution of demographic data, complaints, and comorbidities of the patients included in the study between survivors and non-survivors and the significance levels of the differences between these distributions. Systolic blood pressure (92 vs. 96, p<0.001) mmHg, diastolic blood pressure (70 vs. 71, p<0.001) mmHg, oxygen saturation level (92 vs. 94, p<0.001), and Glasgow coma scale at the time of admission to the emergency medicine clinic (14 vs. 15, p<0.001) were significantly lower in the non-survivors than in the survivors. However, the respiratory rate per minute was significantly higher than it was in the survivors group (25 vs. 24, p<0.001).

**Table 1:** Characteristics of the study patients and significance level of difference between groups

Variables	Non-Survivor (n=627)	Survivor (n=417)	p value
Age, years	66 (58–75)	66 (57–78)	0.942
Fever at admission (°C)	37.0 (36.5–37.2)	37.0 (36.5–37.4)	0.972
Heart Rate at admission, (BPM)	83 (78–90)	83 (74–93)	0.055
Systolic Blood Pressure, at admission (mmHg)	92 (90–98)	96 (92–100)	<0.001
Diastolic Blood Pressure at admission, (mmHg)	70 (67–74)	71 (70–75)	<0.001
Respiratory rate at admission	25(24–26)	24 (22–24)	<0.001
Oxygen saturation at admission (%)	92 (90.0–94.0)	94 (92.0–96.0)	<0.001
GCS at admission	14(13.0–15.0)	15 (15.0–15.0)	<0.001
Hypertension, n(%)	181 (28.9)	139 (33.3)	0.125
Diabetes Mellitus, n (%)	112 (17.9)	90 (21.6)	0.136
CHD, n (%)	79 (12.6)	65 (15.6)	0.171
COPD, n (%)	47 (7.5)	44 (10.6)	0.087
CHF, n (%)	41 (6.5)	39 (9.4)	0.094
Stroke, n (%)	36 (5.7)	35 (8.4)	0.096
CRF, n (%)	22 (3.5)	26 (6.2)	0.039
Asthma, n (%)	23 (3.7)	26 (6.2)	0.055
Hyperlipidemia, n (%)	16 (2.6)	19 (4.6)	0.078

Data are expressed as mean ± standard deviation, median (quartiles) and percentage for categorical variables. BP: Blood Pressure; GCS: Glasgow coma scale; CHD: Coronary heart disease; COPD: Chronic obstructive pulmonary disease; CHF: Chronic heart failure; CRF: Chronic renal failure)

Table 2 presents the median values of lactate and other venous blood gas parameters in the survivors and non-survivors groups and the significance level of the difference between the groups. The lactate (2.6 vs. 1.9, p<0.001) values were significantly higher in the non-survivors group when compared to the survivors group; however, pH (7.35 vs. 7.39, p=0.001), bicarbonate (22.9 vs. 25.3, p<0.001), and base deficit (−2.6 vs 0.5, p<0.001) values were significantly lower.

**Table 2:** Venous blood gas values of the groups and significance level of difference between groups

	Non-Survivor (n=417)	Survivor (n=627)	p value
Lactate, mmol/L	2.6 (1.9–4.4)	1.9 (1.4–2.5)	**<0.001**
pH	7.35 (7.23–7.42)	7.39 (7.34–7.42)	**0.001**
Bicarbonate, mEq/L	22.9 (17.9–26.8)	25.3 (22.8–27.6)	**<0.001**
BaseDeficit, mmol/L	-2.6 (-8.0–1.3)	0.5 (-2.0–2.9)	**<0.001**
Anion Gap, mEq/L	12.2 (9.0–15.5)	12.1 (8.4–15.5)	0.933

Table 3 shows the results of univariate and multivariate logistic regression analyses used to determine the independent risk factors affecting 30-day mortality. Accordingly, gender (OR:1.720; 95% CI 1.294–2.286; p<0.001), systolic blood pressure at admission (OR:0.975; 95% CI 0.960–0.991; p=0.002), the respiratory rate at admission (OR: 1.211; 95% CI 1.128–1.300; p<0.001) and blood gas parameters (OR: 1.220; 95% CI 1.112–1.338; p<0.001) were detected as significant predictors for 30-day mortality.

**Table 3:** Univariate and multivariate regression analyzes of study parameters to predict 30-day mortality

Variables	Univariate		Multivariate	
	
	OR (95% CI)	p value	Adjusted OR(95% CI)	p value
Age, years	1.003 (0.993–1.014)	0.523		
Gender	1.977 (1.526–2.563)	**<0.001**	1.720 (1.294–2.286)	**<0.001**
Heart Rate at Admission, (BPM)	1.003 (0.995–1.010)	0.510		
Systolic Blood Pressure at Admission, (mmHg)	0.961 (0.946–0.975)	**<0.001**	0.975 (0.960–0.991)	**0.002**
Diastolic Blood Pressure at Admission, (mmHg)	0.943 (0.924–0.961)	**<0.001**	0.983 (0.959–1.009)	0.196
Respiratory rate at admission	1.323 (1.239–1.412)	**<0.001**	1.211 (1.128–1.300)	**<0.001**
Oxygen saturation at admission (%)	0.860 (0.829–0.893)	**<0.001**	0.927 (0.887–0.969)	**0.001**
pH	0.021 (0.007–0.066)	**<0.001**	0.201 (0.034–1.171)	0.074
Lactate, mmol/L	1.351 (1.247–1.464)	**<0.001**	1.220 (1.112–1.338)	**<0.001**
Base Deficit, mmol/L	0.951 (0.935–0.967)	**<0.001**	0.996 (0.971–1.022)	0.761
Bicarbonate, mEq/L	0.954 (0.936–0.973)	**<0.001**	0.997 (0.925–1.075)	0.946
Hypertension	1.232 (0.943–1.609)	0.126		
DM	1.266 (0.928–1.726)	0.137		
CHD	1.281 (0.899–1.826)	0.171		
CRF	1.829 (1.022–3.272)	**0.042**		

OR: Odds ratio, Cl: Confidence level; BP: Blood Pressure, DM: Diabetes Mellitus, CHD: Coronary heart disease; CRF: Chronic renal failure

**Figure 1:**
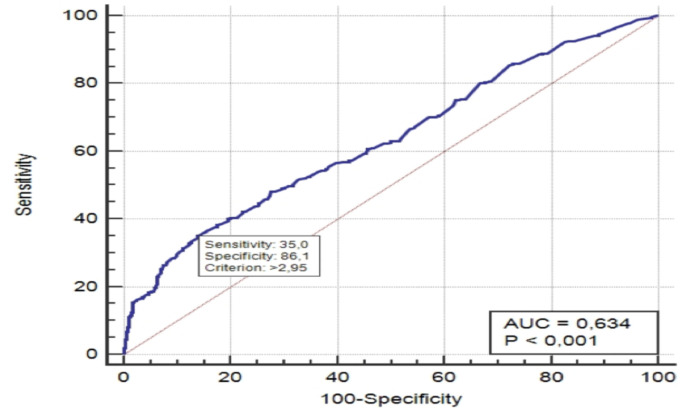
Receiver operating characteristic curve of the admission lactate level for predicting 30-day mortality.

**Figure 2:**
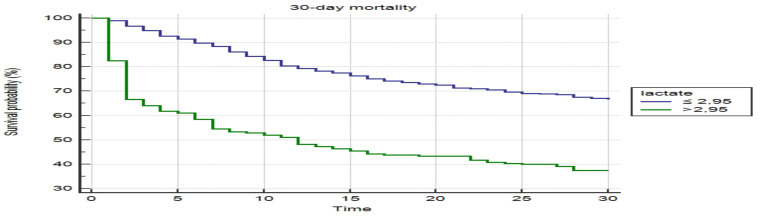
Kaplan-Meier survival curves stratified by the cut-off value (2.95 mg/dL) of admission lactate level to predict 30-day mortality.

When the cut-off value of the lactate level was taken as >2.95 mg/L in the ROC curve to distinguish the non-survivors group from the survivors group, the sensitivity and the specificity were 35.0% and 86.1%, respectively (AUC: 0.634, 95% CI: 0.604–0.663) (Figure 1). On the other hand, the Kaplan-Meier 30-day survival curves of patients with a cut-off value of 2.95 mg/L lactate level are shown in Figure 2. Mortality was significantly higher in patients who had a lactate level above 2.95 mg/L (long-rank test, p<0.001).

## Discussion

The venous blood gas parameters of lactate, pH, bicarbonate, and base deficit in emergency service admissions from the patients were significantly different between non-survivors and survivors. Lactate, which is one of the venous blood gas parameters, was a significant predictor of 30-day mortality. The base deficit and other blood gas parameters including lactate are frequently used in the prognosis of critically ill patients presenting to the emergency medicine clinic. The use of lactate is increasing not only in the emergency department but also in the intensive care units where critically ill patients are followed. The majority of the studies that use lactate to predict a prognosis regard sepsis patients. Studies on patients with critical malignancies are limited.

In their study on 1,129 critical malignancies that they followed in the intensive care unit, Hajjar et al. found that lactate and base deficit analyzed at hours 0 and 24 were significant predictors of mortality (18). In this study, the cutoff value for lactate at hour 0 was 2.3 mmol/L. Similarly, in our study, the lactate level at admission to the emergency department was found as a significant predictor of mortality. Higher lactate levels associate with higher mortality in patients with severe sepsis and septic shock admitted to the emergency medicine clinic (19). Only critical malignancy patients were included in our study. We believe that the cut-off value of 2.95 mmol/L determined for lactate at admission to the emergency clinic may be used for oncological sepsis patients.

One 2016 study investigated the predictivity of lactate in sepsis patients (20). In that retrospective study conducted on 443 patients with severe sepsis and septic shock, the first admission lactate levels were divided into two groups as higher than and lower than 2.5mmol/L. In the study in which 28-day mortality was the primary outcome, the mortality rates of the group with a lactate level above 2.5 mmol/L were determined as 16.5% and the rate of survivors as 5.8%; a statistically significant difference was found (p<0.001) (20). The authors of this study predicted that the cut-off value of 2.5 mmol/L may be used for mortality prediction (20).

A total of 5,440 patients, including 1,837 patients with malignancy, were included in the study in which risk classification was made according to the lactate cut-off values of mortality rates in patients with malignancies admitted to the emergency medicine clinic (21). In this study by Maher et al., the association between 1-day, 3-day, 7-day, and 30-day mortality rates and lactate cut-off values <2 mmol/L, 2–4 mmol/L, and >4 mmol/L was investigated. Although higher lactate values associated with increased mortality in cancer patients, this association was found significant only in 7-day and 30-day mortality. The 30-day mortality was analyzed in this study and critical patients with malignancy were included. We believe that lactate level and 1-day and 3-day mortality rates may be significantly related because only critically ill patients were included in our study. However, further studies are required to show the association between lactate level and 1-day and 3-day mortality rates.

In conclusion, we detected that lactate, which is a blood gas parameter, may be safely used for predicting the 30-day mortality of critical malignancy patients admitted to the emergency medicine clinic. The two centers where we conducted the study are the largest training and research hospitals in Istanbul, and they have most referrals for patients with malignancy. However, this study could have been carried out with a larger multicenter patient population by including other research hospitals in Istanbul. Although this is the most important limitation of the study, this limitation can be dismissed because our study was completed with a large number of patients.
